# Visual and ultrastructural analysis after splitting of recipient’s Descemet membrane during Descemet membrane endothelial keratoplasty

**DOI:** 10.1038/s41433-025-03872-5

**Published:** 2025-05-27

**Authors:** Maximilian Friedrich, Jasper Lind, Hyeck-Soo Son, Timur Mert Yildirim, Gerd Uwe Auffarth, Victor Aristide Augustin

**Affiliations:** Department of Ophthalmology, University of Heidelberg, David J. Apple International Laboratory for Ocular Pathology and International Vision Correction Research Centre (IVCRC), Heidelberg, Germany

**Keywords:** Outcomes research, Corneal diseases, Risk factors

## Abstract

**Background:**

During Descemetorhexis in Descemet Membrane Endothelial Keratoplasty (DMEK), inadvertent lamellar splitting of the recipient’s Descemet membrane (DM) may occur, leaving DM remnants on the posterior corneal surface. This study investigates the influence of lamellar splitting on surgical outcome as well as the histological ultrastructure of split DMs.

**Methods:**

In this prospective, observational, single-centre cohort study 129 eyes of 102 patients were divided into two groups depending on the occurrence of intraoperative splitting. If splitting of the recipient’s DM occurred, the remnants were polished via irrigation/aspiration. The primary outcome was the corrected distance visual acuity (CDVA) four months after DMEK. Secondary outcomes were central corneal thickness (CCT), thinnest corneal thickness (TCT), posterior corneal density (PCD), endothelial cell loss (ECL), and the re-bubbling rate. For histopathological analysis two removed DMs were examined using scanning and transmission electron microscopy.

**Results:**

Intraoperative splitting occurred in 36 eyes (27.9%). The postoperative CDVA in the group with splitting (0.12 ± 0.12 logMAR) did not significantly differ from the group without splitting (0.12 ± 0.12 logMAR; *p* = 0.96). CCT, TCT, PCD, ECL, and the re-bubbling rate also did not significantly differ between both groups (*p* > 0.05). In ultrastructural analysis, the split layer had a thickness of 2 µm and showed an irregular splitting interface.

**Conclusion:**

Inadvertent lamellar splitting of the recipient’s DM during DMEK does not have a significant impact on the visual outcome. Therefore, polishing the DM remnants intraoperatively may address this complication in sufficient manner for optimizing visual outcomes.

## Introduction

Descemet Membrane Endothelial Keratoplasty (DMEK) is the most frequently performed endothelial keratoplasty in the US and Germany to treat endothelial diseases such as Fuchs endothelial corneal dystrophy (FECD) and pseudophakic bullous keratopathy (PBK) [1, 2]. Despite excellent visual results observed after DMEK, there are still complications that may occur perioperatively and undermine the surgical outcomes. Possible postoperative complications include graft detachment with subsequent need for re-bubbling, as well as immunogenic graft rejection or a rise in intraocular pressure [3–5]. Intraoperative complications such as an upside-down graft configuration [6, 7], intraoperative fibrinous reaction [8, 9], or loss of the graft into the vitreous [10] are rare but may deteriorate vision markedly.

During Descemetorhexis, i.e. the removal of the host’s diseased Descemet-Endothelium complex, lamellar splitting of the recipient’s Descemet Membrane (DM) may occur leaving microscopically visible remnants on the posterior corneal surface [11]. A previous study found that accumulations of banded and wide-spaced collagen between the thicker posterior non-banded layer and the thin anterior banded layer of the Descemet membrane may cause lamellar splitting of the DM [11]. The authors suspected a potential impact of diabetes mellitus on the incidence of intraoperative splitting. However, they did not analyse the ultrastructure of the removed Descemet-Endothelium complex histologically via scanning electron microscopy. Furthermore, apart from the re-bubbling rate, which was reported to be similar between eyes with and without splitting, postoperative outcomes such as visual acuity and corneal tomography have yet to be analysed.

This study aimed to analyse whether lamellar splitting of the recipient’s DM during Descemetorhexis for DMEK has a significant impact on the surgical outcome, identify potential systemic risk factors, and analyse the histological ultrastructure of split DMs.

## Materials and methods

We included 129 eyes of 102 patients with FECD or PBK in this prospective, observational, single-centre cohort study. The study flowchart is illustrated in Online Supplementary Fig. 1. To eliminate potential confounding factors related to the patient’s lens or cataract, all included eyes were pseudophakic with monofocal intraocular lenses. Eyes with a history of ocular surgery other than uncomplicated cataract surgery, those with multifocal intraocular lenses, or those with other ocular comorbidities were excluded from the analysis. Additionally, eyes that underwent combined DMEK with cataract surgery (Triple-DMEK) and those that received DMEK due to failed endothelial keratoplasty were also excluded.

This study was approved by the Institutional Review Board/Ethics Committee (ID: S-565/2023) at the Ruprecht-Karls University Heidelberg, Germany, and performed in accordance with the tenets of the Declaration of Helsinki. Informed consent was obtained from all participants.

### Preoperative measurements

All eyes underwent slit-lamp biomicroscopy and presented either with FECD Grade 5 or Grade 6 on the modified Krachmer scale [12] or a PBK. The corrected distance visual acuity (CDVA) was measured for each eye preoperatively in the morning under photopic conditions (320 cd/m²) using an electronic 5-letter per-line chart at 5-meter test distance. All eyes were examined using Scheimpflug tomography (Pentacam AXL, Oculus Optikgeräte, Wetzlar, Germany). The central corneal thickness (CCT), thinnest corneal thickness (TCT), corneal volume, as well as the posterior corneal density (PCD) of the posterior 60 µm were obtained from the *4 Maps Refractive* and *Corneal Densitometry* output. The PCD was measured in grayscale units (GSU).

A detailed medical history was taken including systemic diseases such as diabetes mellitus, arterial hypertension, other cardiovascular diseases, and connective tissue diseases, as well as the nicotine and alcohol history. The nicotine history was documented in pack years, which equals the number of years with a daily consumption of one pack of cigarettes (20 cigarettes). The alcohol consumption was documented as the daily intake of alcohol in grams.

### Surgical procedure

A Nd:YAG laser iridotomy was performed at the 6 and 12 o’clock positions one day prior surgery to minimize the risk of pupillary blockage after DMEK. All surgeries were performed by the same experienced surgeon (V.A.A.) under general anaesthesia. The graft was prepared by the surgeon immediately prior surgery. Graft preparation and DMEK surgery was performed as previously described and was uneventful in all included cases [13].

**Fig. 1:**
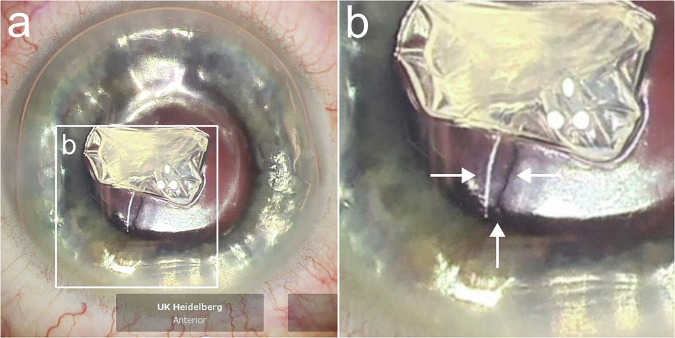
Intraoperative detection of splitting of the recipient’s Descemet Membrane during Descemetorhexis under air with a Descemet incision hook. **a** Overview of the centrally accumulated scraped Descemet-Endothelium complex with splitting inferiorly visible by an altered light reflex. **b** Magnification of the recipient’s split Descemet Membrane highlighted by white arrows.

We performed the scoring and stripping of the host DM (Descemetorhexis, 9 mm) under air by using an inverted Price Endothelial Keratoplasty hook (Geuder AG, Heidelberg, Germany) in all included eyes. Depending on the occurrence of splitting of the recipient’s DM during this step, the eyes were divided into the group with intraoperative splitting or the group without intraoperative splitting. An example for the detection of splitting of the recipient’s DM is shown in Fig. 1. For electron microscopical analysis, the removed DMs were stored in solution with 2.5% glutaraldehyde, 2% paraformaldehyde, and 0.1 M PHEM buffer. The remnants of the DM on the posterior corneal surface were not manually scraped off but polished using a bimanual irrigation/aspiration system in all eyes with splitting. After polishing, no distinct demarcation line was visible anymore.

The graft was injected using a Viscoject-Bio 2.2 injector (Medicel AG, Altenrhein, Switzerland) and unfolded by corneal tapping. After successful unfolding and central positioning of the corneal graft, 100% air tamponade was performed and left for one minute. Then, the anterior chamber was filled with a 20% sulphur hexafluoride (SF6) gas-air-mixture, covering 90% of the horizontal corneal diameter. All patients were postoperatively instructed to maintain a supine position to maximize the bubble graft coverage [14] and reduce complications such as graft detachment or increased intraocular pressure.

### Postoperative measurements

In the postoperative period, we documented all incidents such as graft detachment or increased intraocular pressure. If the graft detached more than 30% of the graft area, a re-bubbling with 20% SF6 gas-air-mixture was performed in local anaesthesia.

The removed Descemet-Endothelium complexes were examined using a scanning electron microscope (Zeiss Leo 1530, Carl Zeiss Microscopy Germany GmbH, Oberkochen, Germany) as well as a transmission electron microscope (Jeol JEM1400, JEOL GmbH, Freising, Germany) to analyse the surface properties of splitting as well as the thickness of the split layer. The examinations were performed by the Electron Microscopy Core Facility (EMCF; RI_00565) at Heidelberg University according to their standard protocols.

Four months after DMEK, we measured the visual acuity again as described above. Additionally, Scheimpflug tomography was performed to measure the postoperative decrease in CCT, TCT, PCD, and corneal volume. The endothelial cell density (ECD) in the central cornea was measured by a specular microscope (CEM-530, NIDEK, Gamagori, Aichi, Japan). The difference between the ECD of the graft before transplantation and ECD four months after DMEK equalled the endothelial cell loss (ECL).

### Statistical analysis

We performed the statistical analysis with SPSS for Windows (Version 29, IBM, Armonk, New York, USA) and R statistical software (Version 4.2.2, R Foundation for Statistical Computing, Vienna, Austria) using the R package “clusrank” [15]. We performed clustered Wilcoxon rank-sum tests using the Datta-Satten method [16] for comparison of metric variables to account for the inclusion of both eyes of a patient in some cases. The primary outcome was the CDVA four months after surgery with a significance level of 0.05. Secondary outcomes were ECL, CCT, TCT, PCD, and the re-bubbling rate. The difference in re-bubbling rate was statistically analysed using a Chi-Square test. The sample size calculation to find a significant difference in CDVA with anticipated means of 0.15 ± 0.1 logarithm of the minimum angle of resolution (logMAR) in the group with intraoperative splitting of the recipient’s Descemet Membrane and 0.1 ± 0.1 logMAR in the group without intraoperative splitting (α = 0.05; β = 0.8) resulted in at least 36 eyes for the splitting group when assuming an incidence of about 1:10 [11].

## Results

The group with DM splitting consisted of 36 eyes (27.9%, 33 patients), while 93 eyes showed no intraoperative splitting. Of the 33 patients with intraoperative splitting, 12 patients had bilateral DMEKs of which three patients showed splitting in both eyes (25.0%). FECD was the predominant indication for DMEK, accounting for 89.1% of all included eyes whereas only 10.9% had PBK as surgical indication. We observed intraoperative splitting in 28.7 and 21.4% of all included eyes with FECD and PBK, respectively. The characteristics of the study patients are shown in Online Supplementary Table 1. In all eyes with splitting, the microscopically visible DM remnants within the central 8 mm zone could be removed with irrigation/aspiration. No further mechanical removal of any stromal strands was necessary.

### Visual acuity

**Fig. 2:**
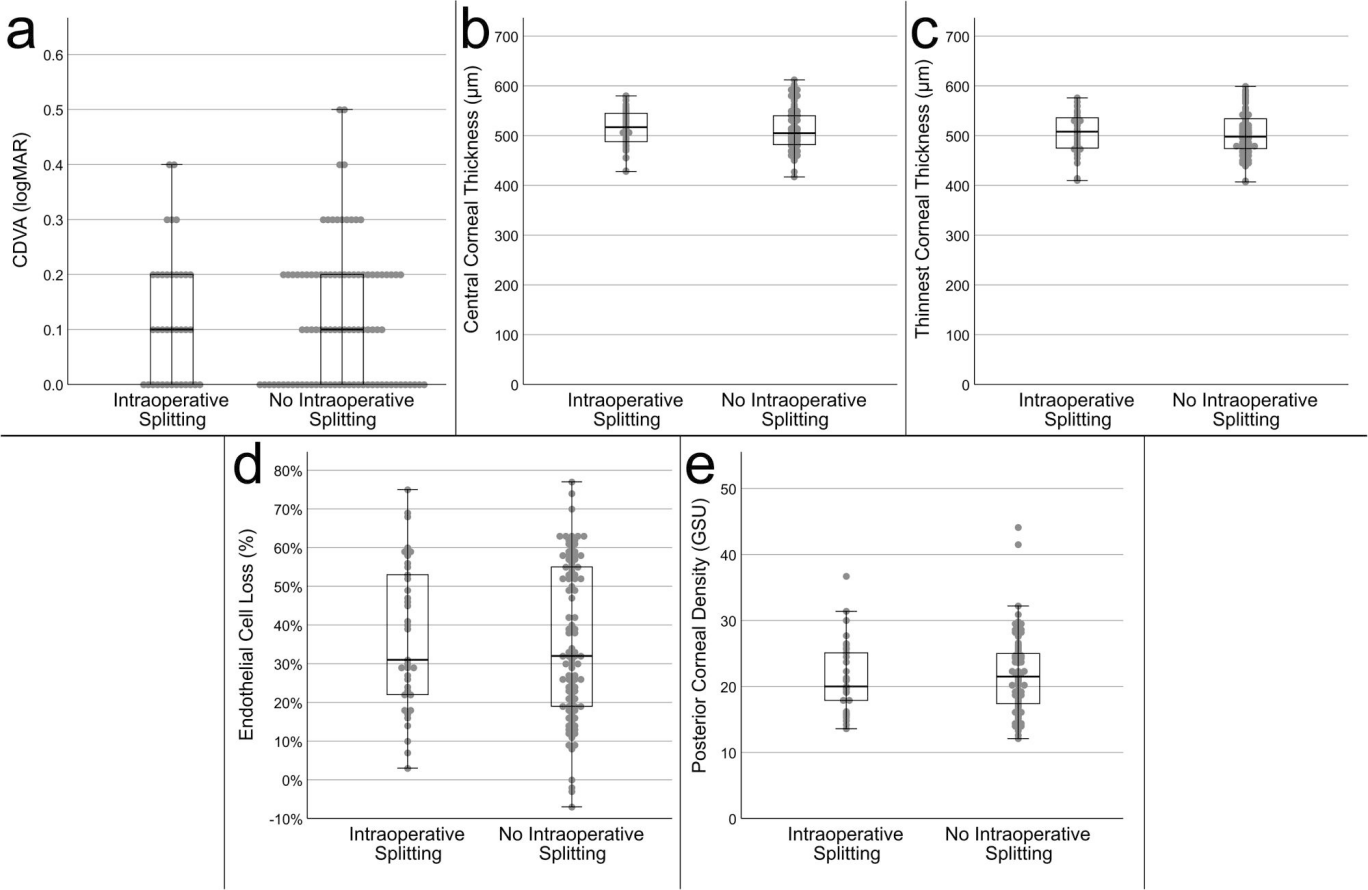
Comparison of outcome parameters four months after Descemet Membrane Endothelial Keratoplasty (DMEK) depending on the intraoperative occurrence of splitting of the recipient’s Descemet Membrane. **a** Corrected distance visual acuity (CDVA) measured in logarithm of the minimum angle of resolution (logMAR). **b** Central corneal thickness measured by Scheimpflug tomography. **c** Thinnest corneal thickness measured by Scheimpflug tomography. **d** Endothelial cell loss calculated by dividing the endothelial cell density measured with a specular microscope four months after DMEK by the preoperative endothelial cell density of the graft. **e** Density values of the posterior 60 µm of the corneal stroma measured by Scheimpflug tomography. GSU Gray scale units.

The CDVA four months after DMEK did not differ statistically significantly between eyes with intraoperative DM splitting (0.12 ± 0.12 logMAR) compared to those without intraoperative splitting (0.12 ± 0.12 logMAR; *p* = 0.955) as shown in Fig. 2a. 94.4% of all cases with intraoperative splitting presented with a postoperative CDVA equal to or better than 0.3 logMAR, compared to 95.7% of all cases without splitting of the recipient’s DM. The preoperative CDVA was inferior in the group without intraoperative splitting (0.56 ± 0.39 logMAR) compared to group with intraoperative splitting (0.42 ± 0.22 logMAR, see Table 1); however, the difference was not statistically significant (*p* = 0.249).

**Table 1:** Descriptive statistics of the examined parameters before and four months after Descemet Membrane Endothelial Keratoplasty (DMEK) depending on the intraoperative occurrence of splitting of the recipient’s Descemet Membrane.

	Intraoperative splitting		No intraoperative splitting	
Parameter	Before DMEK	After DMEK	Before DMEK	After DMEK
Corrected distance visual acuity (Mean ± SD)	0.42 ± 0.22 logMAR	0.12 ± 0.12 logMAR	0.56 ± 0.39 logMAR	0.12 ± 0.12 logMAR
Endothelial cell density (Mean ± SD)	2554.9 ± 184.7 cells/mm²	1614.7 ± 535.5 cells/mm²	2560.7 ± 193.5 cells/mm²	1640.4 ± 522.2 cells/mm²
Central corneal thickness (Mean ± SD)	640.7 ± 88.4 µm	514.6 ± 39.3 µm	647.8 ± 104.8 µm	511.6 ± 41.4 µm
Thinnest corneal thickness (Mean ± SD)	600.7 ± 54.0 µm	507.3 ± 41.0 µm	600.4 ± 86.1 µm	503.2 ± 42.4 µm
Corneal volume (Mean ± SD)	62.9 ± 6.2 mm³	59.3 ± 4.5 mm³	63.2 ± 10.9 mm³	59.8 ± 6.2 mm³
Posterior corneal density (Mean ± SD)	22.2 ± 9.1 GSU	21.2 ± 5.4 GSU	21.3 ± 7.2 GSU	21.8 ± 5.9 GSU

*logMAR* logarithm of the minimum angle of resolution, *GSU* Grayscale units, *SD* Standard deviation.

### Corneal tomography and densitometry

The CCT four months after DMEK did not differ statistically significantly (*p* = 0.41) with a mean of 514.6 ± 39.3 µm and 511.6 ± 41.4 µm in the groups with and without intraoperative splitting, respectively (Fig. 2b). Correspondingly, the TCT four months after DMEK did not differ significantly (*p* = 0.36) with a mean of 507.3 ± 41.0 µm and 503.2 ± 42.4 µm, respectively (Fig. 2c). The postoperative corneal volume was comparable in both groups with a mean volume of 59.3 ± 4.5 mm³ in the group with intraoperative splitting and 59.8 ± 6.2 mm³ in the group without splitting. As shown in Fig. 2e, the PCD four months after DMEK did not significantly differ between the groups with splitting (21.2 ± 5.4 GSU) and without splitting (21.8 ± 5.9 GSU; *p* = 0.56). Preoperatively, the total corneal density also did not differ significantly (*p* = 0.97) between both groups (see Table 1).

### Endothelial cell loss

The mean ECL four months after DMEK was 37.1 ± 19.3% in the group with intraoperative splitting and 35.9 ± 20.0% in the group without splitting (Fig. 2d). The difference in ECL was not statistically significant (*p* = 0.83). The mean pre- and postoperative endothelial cell density is shown in Table 1.

### Complications

In the group with intraoperative splitting of the recipient’s DM, three of 36 eyes (8.3%) presented with a persistent graft detachment and a re-bubbling was performed. In the group without splitting, 16 out of 93 eyes (17.2%) had one re-bubbling and three cases needed two subsequent re-bubblings (3.2%). The re-bubbling rates did not differ statistically significantly (*p* = 0.10). In all cases, we did not observe any rise in intraocular pressure during the postoperative period.

### Systemic diseases and drug abuse

The prevalence of diabetes mellitus was 22.2 and 18.3% in the groups with and without splitting (see Online Supplementary Table 1), respectively, and the difference was not statistically significant (*p* = 0.61). The prevalence of arterial hypertension (66.7 and 66.7%), other cardiovascular diseases (30.6 and 28.0%), and connective tissue diseases (0 and 2.2%) also did not differ significantly between eye with and without intraoperative splitting (*p* > 0.05).

The prevalence of smoking was 22.2% in the group with splitting and 16.1% in the group without splitting (*p* = 0.42). The mean pack years of the smokers were 40.5 ± 43.0 pack years and 41.3 ± 66.6 pack years in the groups with and without splitting, respectively. The prevalence of regular alcohol consumption was 19.4% in both groups (*p* = 1.00). The mean daily alcohol consumption was 51.5 ± 18.1 g/day and 30.3 ± 19.1 g/day in the groups with and without splitting, respectively. However, 36.0% of all regular alcohol consumers did not disclose their daily consumption.

### Microscopy

**Fig. 3:**
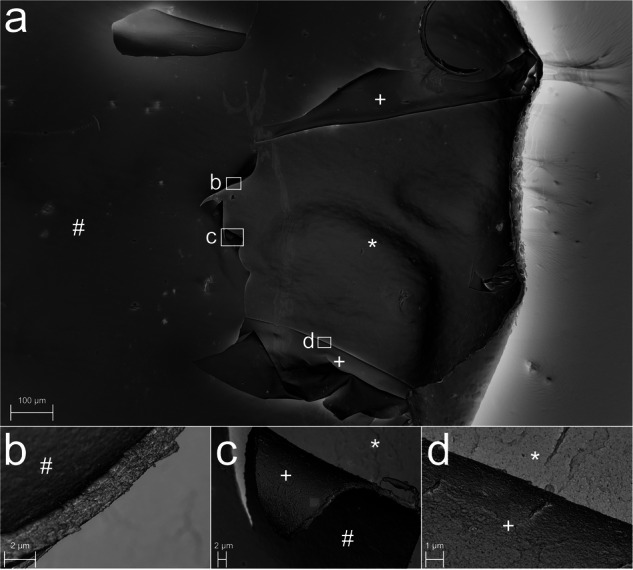
Scanning electron microscopy of an explanted Descemet-Endothelium complex of a recipient’s eye with Fuchs Endothelial Corneal Dystrophy, which showed intraoperative splitting during Descemetorhexis. ^#^Regular Descemet Membrane interface; ^+^Anterior interface of the split Descemet Membrane; ^*^Posterior interface of the split Descemet Membrane. **a** Overview of a region with intraoperative splitting of the recipient’s Descemet Membrane looking from the Descemet interface side. The panels (**b**–**d**) are magnified regions visualized as white rectangles. **b** Magnification of a border between a split and non-split region of the Descemet-Endothelium complex visualizing the thickness of the split layer, which remains as a remnant on the posterior corneal surface. **c** Magnification of the split Descemet Membrane layer curling anteriorly, visualizing the three interfaces of intraoperative splitting. The anterior (+) and posterior (*) interface of the split Descemet Membrane seem more irregular than the regular Descemet Membrane interface (#) after Descemetorhexis. **d** Magnification of the anterior (+) and posterior (*) interface of the split Descemet Membrane showing an irregular surface.

**Fig. 4:**
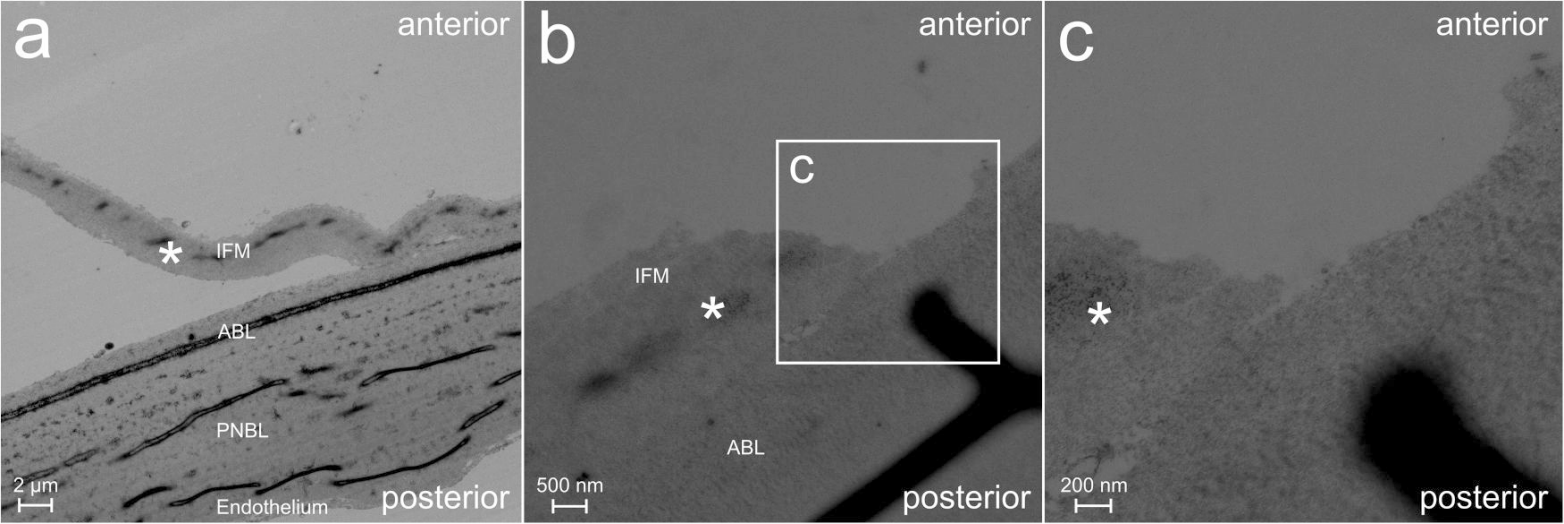
Transmission electron microscopy of an explanted Descemet-Endothelium complex of a recipient’s eye with Fuchs Endothelial Corneal Dystrophy, which showed intraoperative splitting during Descemetorhexis. *Lamellar split layer of Descemet Membrane. Anterior = Anterior side of the Descemet-Endothelium complex. Posterior = Posterior side of the Descemet-Endothelium complex. IFM Interfacial matrix, ABL Anterior banded layer, PNBL Posterior non-banded layer. **a** Cross-sectional view of a region with lamellar splitting without rupture of the split layer. The thickness of the split layer is about 2 µm. **b** Cross-sectional view of a region with lamellar splitting with rupture of the split section. The thickness of the split layer is about 2 µm. **c** Magnification of panel (**b**) showing the irregular border of the split layer.

In scanning electron microscopy, splitting of the recipient’s DM was visualized as a defect on the anterior surface of the removed Descemet-Endothelium complex (Fig. 3a). The border of the region with splitting presented with curled edges, which enabled us to analyse the thickness of the split layer as well as the splitting interface. As shown in Fig. 3b, the thickness of the split layer was about 1.7–2 µm. This is consistent with the images obtained in transmission electron microscopy, which showed a comparable thickness of the split layer (Fig. 4a, b). In terms of the affected layers, splitting occurred most likely in the interfacial matrix, partly with affection of the anterior banded layer (Fig. 4). The posterior non-banded layer did not seem to be affected in lamellar splitting. The splitting interface showed an irregular surface on the anterior and posterior interface surfaces (Fig. 3c, d).

## Discussion

In this study, we demonstrated that inadvertent lamellar splitting of the DM occurs in 27.9% of all primary DMEKs. After polishing the DM remnants, the visual acuity, corneal tomography and density, endothelial cell loss, as well as the re-bubbling rates four months after DMEK did not significantly differ between eyes with intraoperative splitting compared to eyes without splitting. Our analysis suggests that lamellar splitting may be addressed sufficiently via intraoperative polishing without additional surgical manipulation leading to no negative impact on the surgical outcome.

In scanning and transmission electron microscopy, the thickness of the split layer and thus the thickness of the remnants on the posterior corneal surface was about 2 µm. Weller et al. found the remnants to be thicker (4–8 µm) and consisting of the interfacial matrix, the anterior banded layer and partly the posterior non-banded layer [11]. Our samples indicate that lamellar splitting mostly consists of the interfacial matrix and partly of the anterior banded layer, without integration of the posterior non-banded layer. The difference in thickness cannot be explained sufficiently by the surgical techniques as our technique for Descemetorhexis was comparable to their method. Future studies should evaluate whether different forms of splitting occur depending on specific internal or external factors, and whether they have different effects on the surgical outcome, necessitating appropriate surgical measures.

A study by Tourtas et al. showed that a smaller Descemetorhexis, i.e. with a DM-DM overlap between the recipient’s DM and the graft’s DM, resulted in a significantly higher graft detachment and re-bubbling rate compared to DMEKs without a DM-DM interface [17]. This may suggest that DM remnants resulting from intraoperative lamellar splitting increases the re-bubbling rate due to the creation of a DM-DM interface. However, after polishing the remnants with bimanual irrigation-aspiration, the re-bubbling rate did not significantly differ between the groups with and without splitting. This might be due to the sufficient and careful polishing of the remnants or due to the thin split layer as mentioned above compared to the thickness of the whole Descemet-Endothelium complex (around 20 µm). Additionally, if polishing the remnants may not remove them completely, it may regularize the irregular splitting interface that was visualized in our study using scanning electron microscopy. Yet, the graft may also sufficiently attach to the host cornea without polishing the split remnants. Further studies are needed to investigate to which extent polishing has an effect on graft detachment rates.

Previous studies indicated that systemic diseases such as diabetes mellitus may increase the risk for intraoperative splitting due to changes in the collagen composition of the corneal layers [11, 18]. A large multicentric study found diabetes mellitus to be an independent risk factor for graft preparation failure due to tears in the graft [19]. In contrast, we did not find a significantly increased prevalence of diabetes mellitus in the group with intraoperative splitting. Additionally, one study by Schrittenlocher et al. found that other systemic diseases such as heart failure or chronic kidney diseases may increase the risk for DM splitting in donor grafts [20]. However, the prevalence of arterial hypertension, other cardiovascular diseases, and connective tissue diseases did not differ significantly between both groups in our study. We also investigated the alcohol consumption and smoking prevalence in regard to the occurrence of splitting and also did not find a significant difference in both groups. Thus, intraoperative splitting may occur independently of systemic diseases. However, as only 36 eyes with splitting were included in this study, larger study populations may reveal subtle systemic influences on the incidence of intraoperative splitting.

We hypothesized that the PCD as well as the CCT and TCT may be increased and the CDVA may be worse due to DM remnants that were not completely removed but only polished. Yet, we did not find any significant differences in these parameters between both groups. Preoperatively, the PCD may be increased due to corneal oedema or due to the formation of a fibrillar layer [21–23]. Compared to the normative study from Ní Dhubhghaill et al., which found a mean posterior corneal density of 15.70 ± 3.10 GSU in healthy patients [24], both of our groups also showed a higher PCD four months after DMEK. Increased PCD values after DMEK in the early postoperative period were also observed by a previous study [25]. Regarding the postoperative CDVA, one study analysed the effect of graft preparation difficulty including DM splitting on the visual outcome and found no significant differences between an easy or difficult preparation [26]. However, the effect of splitting of the recipient’s DM on the postoperative outcome has not been analysed by another study. Lastly, the ECL did not significantly differ between both groups in our study. Compared to recent reviews describing the ECL after uncomplicated DMEK [3, 4], our results were similar.

In our study, we performed the Descemetorhexis under air to improve visibility of the split remnants via an altered light reflex. Alternatively, Descemetorhexis can be performed under balanced salt solution or viscoelastics. However, split remnants may not be as visible when performing Descemetorhexis under balanced salt solution or viscoelastics. Furthermore, we performed the scoring and stripping of the host DM (Descemetorhexis) under air by using an inverted Price Endothelial Keratoplasty hook in all included eyes [13]. Therefore, it is important to note that different techniques (i.e. using a Descemetorhexis forceps, e.g. Gorovoy DSO Forceps) may result in different incidences of lamellar splitting.

Our study is subject to potential limitations. Since the study was powered specifically for the primary outcome (CDVA), the results related to secondary outcomes and the analysis of potential risk factors should be considered in an exploratory manner and warrant further investigation in a different study population. Additionally, a decline in CDVA can result from various ocular conditions, including cataract or macular degeneration. To mitigate the impact of lenticular causes and other comorbidities on visual acuity, we only included pseudophakic eyes and excluded those with any other ocular comorbidities. Moreover, both eyes of some patients were included in the study, which introduces the possibility of confounding due to interdependence between fellow eyes. To address this, we applied clustered Wilcoxon rank-sum tests to appropriately account for the clustered nature of the data in our statistical analysis [16].

In conclusion, intraoperative lamellar splitting of the recipient’s DM did not have a significant impact on the outcome four months after DMEK when the recipient’s DM remnants were polished intraoperatively. Future studies should investigate the impact of intraoperative splitting on other visual parameters such as contrast sensitivity and straylight depending on the occurrence of intraoperative splitting and analyse whether different forms of splitting may occur due to other factors.

## Summary

### What is already known on this topic:

- During Descemetorhexis in Descemet Membrane Endothelial Keratoplasty (DMEK), inadvertent lamellar splitting of the recipient’s Descemet membrane (DM) may occur, leaving DM remnants on the posterior corneal surface.

### What this study adds:

- Lamellar splitting does not have a significant impact on the visual outcome after polishing the remnants during DMEK.
- In ultrastructural analysis, the split layer had a thickness of 2 µm and showed an irregular splitting interface.
- Lamellar splitting may be sufficiently treated by intraoperative polishing of the Descemet Membrane remnants on the posterior stroma.

## Supplementary information

Online Supplemental Table 1

Online Supplemental Figure 1

Online Supplemental Figure 1

## Data Availability

The datasets generated and analysed during the current study are not publicly available, but are available from the corresponding author on reasonable request.
