# Supplementary material for: Visual and ultrastructural analysis after splitting of recipient’s Descemet membrane during Descemet membrane endothelial keratoplasty
Source: Eye (Lond). 2025 May 27;39(11):2307–13. doi: 10.1038/s41433-025-03872-5 (PMC12274397; doi:10.1038/s41433-025-03872-5)
Supplement: Supplementary file 1 — Online Supplemental Table 1 [file 41433_2025_3872_MOESM1_ESM.docx]

**Online Supplemental Table 1.** Characteristics of all study eyes, depending on the intraoperative occurrence of splitting of the recipient’s Descemet Membrane.

|  | | Intraoperative splitting | No intraoperative splitting |
| --- | --- | --- | --- |
| Eyes (n) | Total | 36 | 93 |
| Gender (n) | Women | 24 | 44 |
|  | Men | 12 | 49 |
| Age (Mean ± SD) | | 74.1 ± 8.3 years | 70.9 ± 10.4 years |
| Eyes (n) | Right | 20 | 57 |
|  | Left | 16 | 36 |
| Corneal disease (n) | FECD | 33 | 82 |
|  | PBK | 3 | 11 |
| Diabetes mellitus (n) | | 8 | 17 |
| Arterial hypertension (n) | | 24 | 62 |
| Other cardiovascular disease (n) | | 11 | 26 |
| Connective tissue disease (n) | | 0 | 2 |
| Active Smokers (n) | | 8 | 15 |
| Regular alcohol consumption (n) | | 7 | 18 |
| Number of re‑bubblings (n) | None | 33 | 74 |
|  | 1 | 3 | 16 |
|  | 2 | 0 | 3 |

*FECD = Fuchs Endothelial Corneal Dystrophy. PBK = Pseudophakic Bullous Keratopathy.*
