# Supplementary material for: Visual and ultrastructural analysis after splitting of recipient’s Descemet membrane during Descemet membrane endothelial keratoplasty
Source: Eye (Lond). 2025 May 27;39(11):2307–13. doi: 10.1038/s41433-025-03872-5 (PMC12274397; doi:10.1038/s41433-025-03872-5)
Supplement: Supplementary file 3 — Online Supplemental Figure 1 [file 41433_2025_3872_MOESM3_ESM.docx]

**Online Supplemental Figure 1.** Illustration of the study methodology. FECD = Fuchs Endothelial Corneal Dystrophy. PBK = Pseudophakic Bullous Keratopathy. DM = Descemet Membrane.
